# ^1^H MR Spectroscopy at 3T for Hepatic Choline Quantification in Healthy Young Women: A Translational Imaging Study with Dietary Correlation

**DOI:** 10.2174/0115734056450482251124081010

**Published:** 2025-11-26

**Authors:** Halima Hawesa, Renad Alghamdi, Hind Allam, Bayader Alfaifi, Norah Alrabiah, Mayar Alghumaiz, Mansour Shanawani, Haya Alshegri, Mahasin G. Hassan

**Affiliations:** 1 Department of Radiological Sciences, College of Health and Rehabilitation Sciences, Princess Nourah bint Abdulrahman University, Riyadh, Saudi Arabia; 2 Department of Radiology, King Abdullah Bin Abdulaziz University Hospital, Riyadh, Saudi Arabia

**Keywords:** Proton Magnetic Resonance Spectroscopy (^1^H-MRS), *In vivo* metabolite quantification, Hepatic choline, Tissue-level biomarkers, Nutrient-sensitive liver conditions, Dietary choline intake, Multiparametric liver MRI, Translational metabolic imaging

## Abstract

**Background:**

Non-invasive biomarkers of liver metabolism are essential for early detection of metabolic alterations. Choline plays a central role in hepatic function, yet its dietary intake and imaging correlates remain underexplored. This study evaluated the feasibility of proton Magnetic Resonance Spectroscopy (^1^H-MRS) at 3T for hepatic choline quantification and examined its correlation with dietary intake in young women, a population at risk of nutrient-sensitive liver conditions.

**Methods:**

In this prospective cohort study, 88 healthy female radiology students (mean age: 21.4 ± 1.8 years) underwent single-voxel ^1^H-MRS of the liver using a 3T Siemens Magnetom Vida scanner. Spectra were acquired with a point-resolved spectroscopy (PRESS) sequence (TR = 2000 ms, TE = 40 ms, voxel size = 20 × 20 × 20 mm^3^), with automated shimming and unsuppressed water referencing. Spectral analysis was performed using LCModel (v6.3), applying quality thresholds (Signal-to-Noise Ratio (SNR) > 5, linewidth < 0.1 ppm, Cramér–Rao Lower Bound (CRLB) < 20%. Hepatic choline concentrations were expressed in Institutional Units (IU). Dietary intake was assessed using a validated Food Frequency Questionnaire (FFQ).

**Results:**

High-quality spectra were consistently obtained (mean SNR: 12.6 ± 3.1; linewidth: 0.048 ± 0.012 ppm). Mean hepatic choline concentration was 4.63 ± 1.21 IU, while mean dietary intake was 29.1 ± 8.7 mg/day. A significant positive correlation was observed (r = 0.555, *p* < 0.001). Regression analysis confirmed dietary intake as a significant predictor (β = 0.56, R^2^ = 0.308, *p* < 0.001).

**Discussion:**

These findings demonstrate that ¹H MRS at 3T provides reproducible hepatic choline quantification and captures meaningful variability linked to dietary intake. The observed correlation highlights the potential of MRS as a translational biomarker of nutrient related liver metabolism. Integrating MRS into multiparametric liver imaging protocols may enhance early detection of metabolic alterations and broaden the scope of non-invasive liver assessment.

**Conclusion:**

^1^H-MRS at 3T is a feasible and reproducible technique for hepatic choline quantification. By measuring metabolites directly in the liver at their site of production, rather than in circulation, where concentrations may be altered, MRS provides physiologically relevant insights into nutrient-related hepatic metabolism. Its correlation with dietary intake highlights its potential as a translational imaging biomarker for early detection and risk stratification of nutrient-sensitive liver conditions.

## 1 INTRODUCTION

The escalating burden of Non-Alcoholic Fatty Liver Disease (NAFLD) highlights a critical gap in clinical practice: the lack of non-invasive tools that quantify hepatic metabolism directly at the tissue level. Non-Alcoholic Fatty Liver Disease (NAFLD) and related metabolic disorders are rising globally, highlighting the need for non-invasive biomarkers of hepatic metabolism. Choline is a vital nutrient involved in lipid transport, gene regulation, cellular signalling, and antioxidant defence. Its metabolites, including phosphatidylcholine and betaine, support membrane integrity, methylation, and detoxi-fication-functions disrupted in NAFLD and hepatic steatosis [1-6].

Dietary intake is the primary source of choline, with eggs, meats, fish, dairy, and grains as major contributors [7-10]. Cooking methods and food matrices influence absorption [10, 11]. Despite its importance, global intake remains suboptimal [12], especially in regions such as Saudi Arabia, where dietary transitions among young adults may reduce consumption of choline-rich foods. This is particularly concerning for women of reproductive age, whose physiological demand increases during pregnancy, lactation, and hormonal cycling [13-16]. Over 90% of women in this group fail to meet the Adequate Intake (AI) for choline, often due to reduced consumption of eggs, liver, and meat-especially among those following plant-based or low-fat diets [8, 14, 16]. Wallace *et al*. reported that insufficient intake may elevate liver enzymes and impair methylation, increasing the risk of hepatic dysfunction and adverse pregnancy outcomes [13, 14]. Hepatic choline levels may also be influenced by age, BMI, and caloric intake-factors affecting liver metabolism and nutrient absorption. These variables were included as covariates to isolate dietary effects.

In Europe, average intake ranges from 250–400 mg/day, below the AI of 425 mg for women and 550 mg for men. Similar trends are seen globally, with processed and plant-based diets reducing choline-rich food consumption [14]. In Saudi Arabia, emerging data suggest a decline in intake among university students, particularly among females. Combined with sedentary lifestyles, micronutrient deficiencies, and rising obesity rates [17], this positions young Saudi women as a vulnerable group for early hepatic disease. These patterns justify investigating nutrient-related metabolic markers in this population.

Conventional choline quantification relies on biochemical assays or chromatography. Enzymatic methods use choline oxidase for rapid detection, while LC-MS/MS offers high sensitivity and specificity (> 95%) for choline derivatives [11, 18]. These techniques, however, require invasive sampling and complex preparation. In contrast, ^1^H-MRS enables non-invasive, *in vivo* quantification of choline-containing compounds directly in the liver, providing real-time metabolic insights at the site of production rather than in circulation, where concentrations may be altered by transport or clearance.

In ^1^H-MRS, Phosphocholine (PCho) and Glycerophospho-choline (GPC) resonate near 3.2 ppm. Spectral-fitting software, such as LCModel, deconvolutes overlapping signals using a predefined basis set. In liver MRS, the 3.2 ppm peak reflects total choline, indicating membrane turnover and phospholipid metabolism. Values are expressed in Institutional Units (IU), representing signal intensity ratios relative to unsuppressed water. Though not directly convertible to mmol/kg, IU values allow standardized comparison across subjects and scanners. Single-voxel techniques such as PRESS and Stimulated Echo Acquisition Mode (STEAM) are conventional in hepatic MRS. PRESS offers higher SNR and better localization, while STEAM allows shorter echo times and improved resolution. Each has trade-offs in sensitivity and spectral quality [19-21]. Multiparametric liver imaging increasingly integrates MRS with PDFF and MR elastography. Combined protocols show diagnostic accuracies of 85–92% for liver fibrosis, steatosis, and iron overload [22, 23].

MRS adds metabolic insight, capturing early stress before structural damage. Altered choline metabolism is linked to inflammation, fibrosis, and impaired lipid export. Recent surveys among Saudi university students report average choline intake below 300 mg/day, with many failing to meet AI thresholds, especially among females [24]. This reinforces the relevance of hepatic choline quantification for preventive screening. Gut microbiota, known to modulate choline absorption, may further influence hepatic levels and should be considered in future studies. Establishing reproducible reference values is essential for clinical translation and early detection of metabolic alterations [7, 19].

Given these dietary and lifestyle shifts, this study investigates the feasibility of ^1^H-MRS at 3T for hepatic choline quantification and its correlation with dietary intake in young Saudi women. We hypothesize that measurable differences in hepatic choline reflect dietary variation, supporting ^1^H-MRS as a translational metabolic imaging tool that provides physiologically relevant information directly from the liver.

## 2 METHODOLOGY

### 2.1 Study Design and Ethical Approval

This prospective cross-sectional study was conducted from January to June 2024 at King Abdullah bin Abdulaziz University Hospital (KAAUH), Riyadh. The aim was to assess the association between dietary choline intake and hepatic choline levels in female students from the Diagnostic Radiology Program at Princess Nourah bint Abdulrahman University (PNU). KAAUH was selected for its advanced 3T MRI infrastructure and proximity to PNU. Recruitment from a single academic program ensured demographic homogeneity and logistical feasibility.

### 2.2 Sample Size Justification

Using G*Power (v3.1) [25], a priori power analysis (r = 0.40, α = 0.05, power = 0.80) indicated a minimum of 66 participants. To account for data loss, 88 were recruited. After excluding 6 datasets due to poor spectral quality, 82 datasets were analyzed, exceeding the required threshold. The study was approved by PNU’s IRB (Log No. 23 0226), and written informed consent was obtained. All procedures followed the Declaration of Helsinki and Strengthening the Reporting of Observational Studies in Epidemiology (STROBE) guidelines.

### 2.3 Participants

Healthy female students aged 18–25 were recruited via campus advertisements and email invitations. Inclusion criteria: non-pregnant, non-smoking, and free from hepatic, metabolic, gastrointestinal, or neurological disorders. These exclusions minimized confounding influences on hepatic metabolism. MRI contraindications (*e.g.*, implants, claustro-phobia) were also excluded. Participants fasted for ≥ 4 hours before scanning. Height, weight, and BMI were recorded on imaging day.

### 2.4 MRI and MRS Acquisition

Imaging was performed on a 3T Siemens Magnetom Vida scanner using an 18-channel phased array body coil. Participants were positioned supine, head-first, with motion minimized using foam supports.

For voxel localization, a T2-weighted three-plane HASTE sequence was acquired (acquisition time < 25 seconds). Single-voxel ^1^H-MRS was then performed using a PRESS sequence (voxel size 20 × 20 × 20 mm^3^; TR 2000 ms; TE 40 ms; spectral width 2000 Hz; 1024 data points; 64 signal averages; water suppression bandwidth 50 Hz). The voxel was positioned in the right hepatic lobe, carefully avoiding major vessels and bile ducts. Automated second-order shimming was applied to optimize field homogeneity. In addition, unsuppressed water spectra were acquired for reference and subsequent quantification.

### 2.5 Spectral Processing and Quality Control

Spectra were analyzed using LCModel (v6.3) with a vendor-supplied basis set. The choline peak at 3.2 ppm was quantified relative to the unsuppressed water spectrum, with metabolite levels expressed in Institutional Units (IU). LCModel was run with water scaling, and eddy current correction was applied using the unsuppressed water signal. Automatic phase and baseline corrections were performed. Spectra were excluded if SNR < 5, FWHM > 0.1 ppm, or CRLB > 20%. Reproducibility was assessed in 20 reanalyzed spectra, yielding an intraclass correlation coefficient (ICC) of 0.92. Bland–Altman plots evaluated agreement.

### 2.6 Dietary Choline Intake Assessment

Dietary intake was assessed using a validated semi-quantitative Food Frequency Questionnaire (FFQ) [12] that covered the frequency and portion size of choline-rich foods (*e.g.*, eggs, dairy, grains, fish, legumes, leafy vegetables). Nutrient values were derived from the peer-reviewed study “Concentrations of choline-containing compounds and betaine in common foods,” which was developed in collaboration with the USDA [11]. Weekly intake was calculated by multiplying each item's frequency by its nutrient content and summing across items. Preparation methods and seasonal availability were recorded to improve accuracy.

### 2.7 Data Collection and Statistical Analysis

For each participant, hepatic choline levels obtained from MRS and dietary intake derived from the FFQ were compiled into a single dataset. Statistical analyses were performed using SPSS version 25 (IBM Corp., Armonk, NY, USA). Descriptive statistics were used to summarize participant characteristics. The Shapiro–Wilk test was applied to assess the normality of distributions. Associations between hepatic choline and dietary intake were first explored using Pearson correlation. Univariate regression was then performed to evaluate direct associations, followed by multivariate regression models adjusting for age, BMI, and total caloric intake. Reproducibility of MRS measurements was assessed using Bland–Altman plots. Results are presented as regression coefficients with 95% confidence intervals (CIs), and *p*-values < 0.05 were considered statistically significant.

## 3 RESULTS

Eighty-eight female undergraduates (mean age: 21.4 ± 1.8 years; BMI: 23.1 ± 2.9 kg/m^2^) completed MRI/MRS and dietary assessment. Six spectra were excluded due to poor quality (linewidth > 0.1 ppm or CRLB > 20%), yielding 82 analyzable datasets. Baseline demographic and anthropometric characteristics are summarized in Table **1**.

Spectral quality was consistently high, with a mean SNR of 12.6 ± 3.1 and a mean linewidth of 0.048 ± 0.012 ppm. The CRLB for choline was below 15% in 92% of spectra, confirming reliable quantification. Bland–Altman analysis of 20 repeated scans demonstrated minimal bias with narrow limits of agreement, while inter-observer reproducibility was excellent (ICC = 0.92). Representative voxel placement and a typical spectrum are shown in Figs. (**1** and **2**).

Summary statistics for hepatic choline concentration and dietary intake are presented in Table **2**. A significant positive correlation was observed between dietary intake and hepatic choline concentration (r = 0.555, 95% CI: 0.39–0.69, *p* < 0.001), as illustrated in Fig. (**3**). Dietary choline intake significantly predicted hepatic choline concentration, explaining 31% of the variance (R^2^ = 0.308). This association remained robust after adjustment for age, BMI, and caloric intake Table **3**.

No reference diagnostic method (*e.g.*, biopsy, PDFF, serum choline) was included; therefore, sensitivity and specificity could not be determined. Nevertheless, the observed correlation supports the physiological relevance of MRS-derived hepatic choline. Future studies should incorporate validated endpoints to establish diagnostic thresholds and assess performance.

## 4 DISCUSSION

Choline deficiency, even in the presence of adequate folate and vitamin B12, has been linked to metabolic complications such as NAFLD. Wallace *et al*. [14] reported that low choline intake elevates liver enzymes and circulating creatine, indicating hepatic stress and muscle damage-underscoring choline’s essential role in liver function and cellular integrity. In this study, we observed a significant positive correlation between dietary choline intake and hepatic choline concentration measured by ^1^H-MRS at 3T, supporting the hypothesis that dietary choline influences hepatic metabolism and aligning with Wallace *et al*.’s findings.

^1^H-MRS enables non-invasive quantification of choline-containing compounds such as phosphocholine and glycero-phosphocholine, providing insight into membrane turnover and hepatocellular activity. Unlike biochemical assays, ^1^H-MRS detects early metabolic changes before structural damage occurs [19-21].

Our findings are consistent with Van Parys *et al*. [12], who demonstrated that higher intake of choline-rich foods increased hepatic choline levels, though they also reported a concomitant rise in hepatic fat. This highlights the importance of balanced dietary strategies that ensure adequate choline intake without promoting lipid accumulation.

Beyond hepatic metabolism, choline plays a critical role in neurodevelopment. Dymek *et al*. [13] emphasized increased maternal demand during pregnancy and lactation, with inadequate intake potentially impairing fetal brain development. Although our cohort did not include pregnant women, the findings remain relevant to young women of reproductive age, who may be at risk of insufficient intake. Korsmo *et al*. [15] similarly noted the global under-recognition of choline’s importance for maternal and general health.

From an imaging perspective, our study confirms the feasibility of ^1^H-MRS at 3T for hepatic choline quantification in healthy young adults. Spectral reproducibility, along with acceptable SNR and linewidth, supports its reliability. Prior studies have shown that ^1^H-MRS complements MRI-based fat quantification and elastography in NAFLD assessment [26-28]. Multiparametric MRI protocols now integrate fat, iron, and fibrosis biomarkers [22], and McDonald *et al*. [23] validated Liver Multi-Scan against histological measures. Our findings extend this by demonstrating that ^1^H-MRS can capture nutrient-related metabolic variability, adding value to clinical and research workflows.

Although we did not include a reference diagnostic method, such as liver biopsy, serum choline, or PDFF, these are commonly used in hepatic evaluation. PDFF is a validated MRI biomarker for liver fat and widely used in NAFLD screening [29]. Compared to PDFF, ^1^H-MRS directly quantifies choline metabolites, reflecting membrane turnover rather than lipid content. While ^1^H-MRS requires longer acquisition and specialized processing, it offers unique metabolic insights. Sensitivity and specificity for detecting choline deficiency could not be determined in this study. Future research should compare ^1^H-MRS with PDFF, elastography, and serum biomarkers to establish diagnostic performance [30].

## 5 STRENGTHS AND LIMITATIONS

A key strength of this study is the integration of validated dietary assessment with advanced imaging, providing a multidimensional view of hepatic metabolism. The cross-sectional design, however, precludes causal inference, and reliance on self-reported dietary intake may introduce recall bias. Single-voxel sampling could limit representation of regional metabolic heterogeneity, and the findings may not generalize beyond young Saudi women. The absence of a reference diagnostic method (*e.g.*, biopsy, PDFF, serum choline) also prevented estimation of sensitivity and specificity.

It is important to clarify that metabolite quantification by *in vivo* ^1^H MRS is fundamentally different from diagnostic test validation. In spectroscopy studies, reliability is established through spectral quality control parameters such as SNR, linewidth, and CRLB, rather than by sensitivity or specificity against serum assays or biopsy. Crucially, ^1^H-MRS quantifies metabolites directly at their site of production *in vivo* (*e.g.*, liver, brain), capturing concentrations in the tissue microenvironment where they are synthesized and utilized. By contrast, serum assays or biopsy samples reflect systemic or downstream concentrations that may be altered by transport, metabolism, or clearance. Thus, ^1^H-MRS provides a physiologically more relevant measure of local metabolism that cannot be directly compared with blood or biopsy values [26, 31-34].

Additionally, expressing hepatic choline concentration in “institutional units” (IU), while standardized within the LCModel framework [19], limits direct comparison with studies reporting absolute concentrations (*e.g.*, mmol/kg wet weight). Finally, gut microbiota composition and related dietary factors (*e.g.*, fibre, probiotics), known to influence choline bioavailability [7, 10], were not assessed and may represent unmeasured confounders.

### 5.1 Clinical Relevance

This study demonstrates that ^1^H-MRS at 3T can non-invasively quantify hepatic choline, offering a reproducible biomarker of liver metabolism. By capturing nutrient-related variability, MRS may support early detection of metabolic changes and complement existing MRI techniques. These findings reinforce its potential integration into multiparametric liver imaging, particularly for populations at risk of dietary insufficiency.

However, clinical implementation pathways remain to be defined. Reference thresholds for hepatic choline concentration-especially in relation to dietary intake and NAFLD risk-have not yet been established [31, 32]. Furthermore, the complementary role of MRS-derived choline alongside validated biomarkers such as PDFF and MR elastography requires further evaluation [29, 30]. Future studies should aim to establish clinically actionable cut-offs and assess how MRS can enhance diagnostic accuracy, risk stratification, and treatment monitoring in hepatology practice.

## CONCLUSION

This study confirms the feasibility and reproducibility of ^1^H-MRS at 3T for non-invasive hepatic choline quantification in healthy young women. High-quality spectra were consistently obtained, with robust signal-to-noise ratios, narrow linewidths, and reproducibility validated by interobserver agreement and Bland–Altman analysis. Hepatic choline levels showed a significant positive correlation with dietary intake, explaining approximately one-third of the variance. These findings demonstrate that MRS can capture nutrient-related variability in hepatic metabolism and may complement established MRI tools such as PDFF and MR elastography. While the cross-sectional design and reliance on self-reported dietary data limit causal inference, integrating advanced imaging with validated nutritional assessment provides a strong foundation for future research. Larger, longitudinal, and multiethnic studies are needed to establish reference thresholds, validate clinical utility, and explore the role of MRS in guiding dietary or therapeutic interventions.

Importantly, ^1^H-MRS quantifies metabolites directly in the liver at their site of production, providing physiologically relevant insights that cannot be obtained from serum or biopsy, which reflect downstream or altered concentrations. In summary, ^1^H-MRS at 3T offers a reproducible, non-invasive, and translationally relevant approach to hepatic choline quantification, supporting its integration into multiparametric liver imaging for early detection, risk stratification, and personalized management of nutrient-sensitive liver disease.

## Figures and Tables

**Fig. (1):**
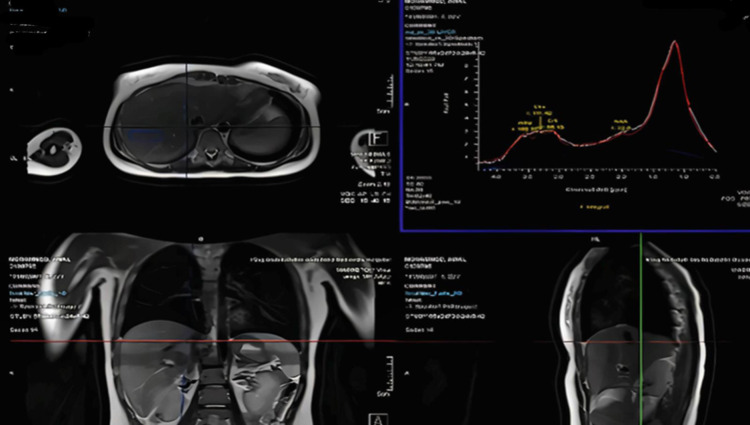
Axial T2-weighted MR image showing voxel placement for hepatic ^1^H-MRS. A single voxel (20 × 20 × 20 mm^3^, blue box) is positioned in the right hepatic lobe, carefully avoiding major vessels and bile ducts. A blue arrow indicates the voxel location, with adjacent anatomical landmarks such as the portal vein and liver capsule visible for orientation.

**Fig. (2):**
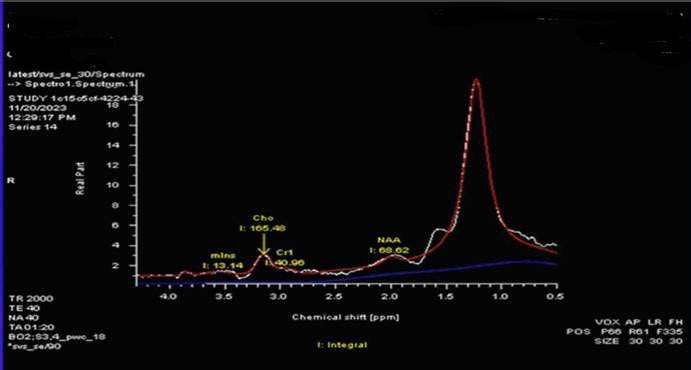
Representative hepatic ^1^H-MR spectrum from the voxel shown in Fig. (**1**), acquired using a PRESS sequence (TE = 40 ms). The x-axis represents chemical shift (ppm) and the y-axis represents signal intensity. The Choline (Cho) peak at 3.2 ppm was quantified using LCModel.

**Fig. (3):**
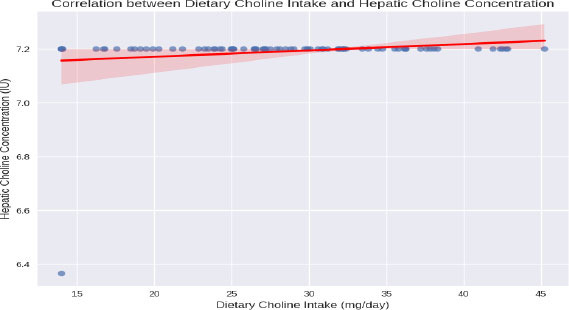
Scatterplot of dietary choline intake (x-axis, mg/day) versus hepatic choline concentration (y-axis, IU). The regression line is shown with 95% confidence interval shading.

**Table 1:** Demographic and nutritional characteristics of study participants (n = 82). Values are expressed as mean ± Standard Deviation (SD) with observed ranges in parentheses.

**Variable**	**Mean ± SD**	**Range**
Age (years)	21.4 ± 1.8	18–25
BMI (kg/m^2^)	23.1 ± 2.9	18.5–29.4
Daily caloric intake (kcal)	1,982 ± 412	1,420–2,780
Dietary choline intake (mg/day)	29.1 ± 8.7	14–52

**Abbreviations:** BMI, body mass index; kcal, kilocalories.

**Table 2:** Mean hepatic choline concentration and dietary choline intake in healthy young women (n = 82). Values are expressed as mean ± Standard Deviation (SD) with 95% Confidence Intervals (CI). Hepatic choline is reported in Institutional Units (IU) derived from ^1^H-MRS quantification.

**Variable**	**Mean ± SD**	**95% CI**
Hepatic choline (IU)	4.63 ± 1.21	4.36–4.90
Dietary choline (mg/day)	29.1 ± 8.7	27.2–31.0

**Table 3:** Multivariate linear regression analysis of dietary choline intake as a predictor of hepatic choline concentration measured by ^1^H-MRS at 3T. The model was adjusted for age, Body Mass Index (BMI), and total caloric intake.

**Predictor**	**β**	**SE**	**95% CI**	***p*-value**
Dietary choline intake	0.56	0.09	0.36–0.72	<0.001

**Abbreviations:** β, regression coefficient; SE, standard error; CI, confidence interval.

## Data Availability

The datasets generated and analyzed during the current study are not publicly available due to participant confidentiality, but are available from the corresponding author [H.H] on reasonable request.

## LIST OF ABBREVIATIONS

^1^H MRS: Proton Magnetic Resonance Spectroscopy
AI: Adequate Intake
ANOVA: Analysis of Variance
BMI: Body Mass Index
CI: Confidence Interval
CRLB: Cramér–Rao Lower Bound
FFQ: Food Frequency Questionnaire
FWHM: Full Width at Half Maximum
ICC: Intraclass Correlation Coefficient
IU: Institutional Units
KAAUH: King Abdullah bin Abdulaziz University Hospital
LCModel: Linear Combination Model
MRI: Magnetic Resonance Imaging
MRS: Magnetic Resonance Spectroscopy
NAFLD: Non Alcoholic Fatty Liver Disease
PDFF: Proton Density Fat Fraction
PCho: Phosphocholine
PNU: Princess Nourah bint Abdulrahman University
PRESS: Point Resolved Spectroscopy
SNR: Signal to Noise Ratio
SPSS: Statistical Package for the Social Sciences
STEAM: Stimulated Echo Acquisition Mode
STROBE: Strengthening the Reporting of Observational Studies in Epidemiology
TE: Echo Time
TR: Repetition Time
USDA: United States Department of Agriculture

## STANDARDS OF REPORTING

STROBE guidelines were followed.
